# A Core Metabolome Response of Maize Leaves Subjected to Long-Duration Abiotic Stresses

**DOI:** 10.3390/metabo11110797

**Published:** 2021-11-22

**Authors:** Jaya Joshi, Ghulam Hasnain, Taylor Logue, Madeline Lynch, Shan Wu, Jiahn-Chou Guan, Saleh Alseekh, Alisdair R. Fernie, Andrew D. Hanson, Donald R. McCarty

**Affiliations:** 1 Horticultural Sciences Department, University of Florida, Gainesville, FL 32611, USA; jayajoshi20@gmail.com (J.J.); logue.taylor@yahoo.com (T.L.); madelinelynch0428@gmail.com (M.L.); wus@ufl.edu (S.W.); guanjc@ufl.edu (J.-C.G.); adha@ufl.edu (A.D.H.); 2 Department of Biology, University of North Georgia, Oakwood, GA 30566, USA; Ghulam.Hasnain@ung.edu; 3 Max-Planck-Institute of Molecular Plant Physiology, 14476 Potsdam-Golm, Germany; Alseekh@mpimp-golm.mpg.de (S.A.); Fernie@mpimp-golm.mpg.de (A.R.F.); 4 Center for Plant Systems Biology, 4000 Plovdiv, Bulgaria

**Keywords:** drought, salinity, heat stress, metabolomics, RNA-seq

## Abstract

Abiotic stresses reduce crop growth and yield in part by disrupting metabolic homeostasis and triggering responses that change the metabolome. Experiments designed to understand the mechanisms underlying these metabolomic responses have usually not used agriculturally relevant stress regimes. We therefore subjected maize plants to drought, salt, or heat stresses that mimic field conditions and analyzed leaf responses at metabolome and transcriptome levels. Shared features of stress metabolomes included synthesis of raffinose, a compatible solute implicated in tolerance to dehydration. In addition, a marked accumulation of amino acids including proline, arginine, and γ-aminobutyrate combined with depletion of key glycolysis and tricarboxylic acid cycle intermediates indicated a shift in balance of carbon and nitrogen metabolism in stressed leaves. Involvement of the γ-aminobutyrate shunt in this process is consistent with its previously proposed role as a workaround for stress-induced thiamin-deficiency. Although convergent metabolome shifts were correlated with gene expression changes in affected pathways, patterns of differential gene regulation induced by the three stresses indicated distinct signaling mechanisms highlighting the plasticity of plant metabolic responses to abiotic stress.

## 1. Introduction

Agriculturally important abiotic stresses for crop plants include heat, drought, and salinity (salt). These stresses are expected to become increasingly prevalent worldwide due to climate change [1]. Photosynthesizing leaves are particularly sensitive to these stresses because the capacity for uptake of CO_2_ through leaf stomatal pores must be balanced against the rate of water loss via transpiration. For that reason, the physiological impacts of heat, drought, and salt stress are likely to differ in fundamental ways. Under heat stress, transpiration contributes to leaf cooling provided that transport of water from the roots can be maintained, whereas under drought stress, transpiration and gas exchange are curtailed by stomatal closure to prevent dehydration [2,3]. Under salt stress, transpiring leaves accumulate high concentrations of salt, creating osmotic stress as well as ionic imbalances [4]. Thus, while heat, drought, and salt stresses have distinct impacts on photosynthetic metabolism, they overlap and interact in important ways [5,6]. All include a likelihood that leaf cells of stressed plants will experience lowered water potentials.

Metabolites broadly implicated in plant abiotic stress tolerance include the protein amino acids proline and arginine and the non-protein amino acids ornithine and γ-aminobutyric acid (GABA) [4,5,7]. Proline functions principally as a protective osmolyte [8,9] and arginine and ornithine are precursors of proline and polyamines [10]. The multifaceted roles of GABA and the GABA shunt in abiotic stresses are not yet fully resolved [11,12]. GABA is both a key intermediary between the tricarboxylic acid (TCA) cycle and nitrogen assimilation pathways and a signaling molecule [12]. Moreover, because the GABA shunt bypasses the thiamin-dependent 2-oxoglutarate dehydrogenase reaction of the TCA cycle, it provides a potential workaround for stress-induced functional thiamin deficiency [13,14,15]. Raffinose series sugars confer tolerance to dehydration in seeds and other organs by functioning as compatible osmolytes and antioxidants [16,17,18].

Gene expression changes induced by stress [19,20,21,22] fall into four broadly defined functional categories: (1) stress-induced transcription factors; (2) genes involved in posttranslational modifications including phosphorylation and dephosphorylation; (3) chaperonins; and (4) genes involved in production of osomoprotectants and stress metabolites. Genes in the latter category are a focus of this study.

Despite extensive research on abiotic stress responses, the physiological implications of stress-induced changes in metabolism and the signaling mechanisms that reshape the metabolome under stress remain poorly understood. One limitation is that experiments designed to understand the mechanisms underlying these metabolomic responses have usually not used agriculturally relevant stress regimes. Treatments are typically imposed abruptly and for a short duration (<24 h)–conditions that rarely occur in field environments [19,20,21,22].

Here we analyze leaf metabolomes and transcriptomes of maize plants subjected to long-term heat, drought, or salt stresses carefully designed to mimic realistic field environments. Our results reveal a suite of metabolome changes that are common to all three abiotic stress treatments. These core changes in the metabolome are correlated with gene expression changes in associated metabolic pathways. However, the convergent changes in heat, drought, and salt stress metabolomes, respectively, arise from distinct signaling mechanisms that differentially target genes in key pathways, highlighting the plasticity of plant stress responses.

## 2. Results

### 2.1. Changes in Central Metabolism in Response to Long-Term Drought, Heat, or Salt Stress

In agricultural environments, crop plants frequently encounter stress conditions of prolonged duration (e.g., a week or longer) that reduce growth and biomass yield. Although progress has been made in understanding correlations between stresses and plant metabolomes [23,24], experiments typically have not used agriculturally realistic stress conditions. To quantify effects of realistic stress environments on metabolic homeostasis of maize leaves, we analyzed leaf metabolomes of B73 inbred plants grown under long-term, non-lethal heat, drought, or salt stress treatments. As detailed in Materials and Methods, drought stress imposed by withholding water intensified gradually over a two-week period to mimic a natural stress environment. Salt stress treatments were carefully designed based on classical principles to avoid confounding effects of leaching of soil nutrients, calcium deficiency, and other types of inadvertent damage. Leaf metabolomes and transcriptomes of stressed plants were then compared to those of unstressed control plants. At least six biological replicates were used for metabolite analyses and three biological replicates were analyzed by RNA-seq.

### 2.2. Shared Features of Stress Metabolomes

**Figure 1:**
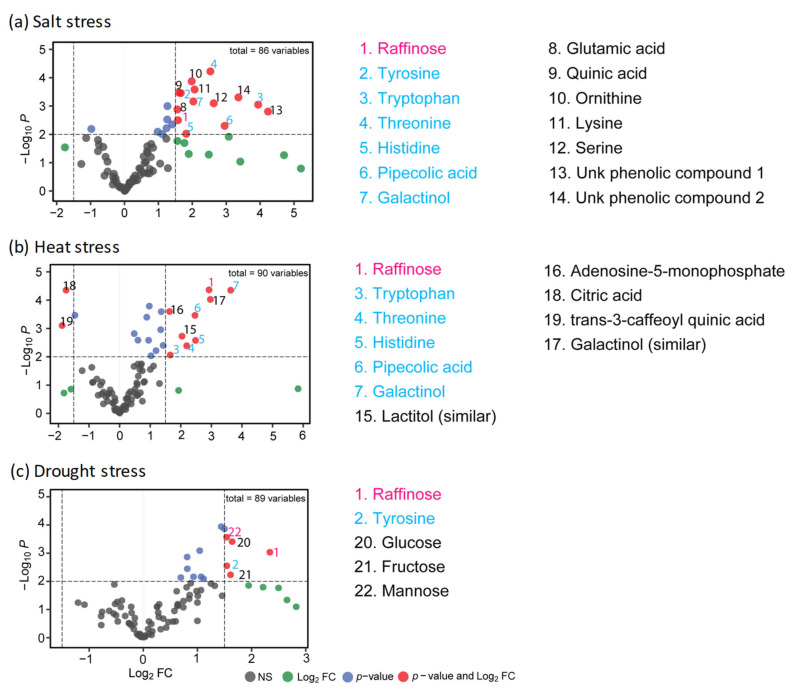
Enhanced volcano plots showing significantly changed metabolites in (**a**) salt stress, (**b**) heat stress, and (**c**) drought stress conditions. Volcano plots are represented with Log_2_ fold change (FC) on *x*-axis and −Log_10_ *p*-value on the *y*-axis. Each point represents a different metabolite. Circle colors indicate classification of metabolites based on Log_2_ FC absolute value using a cutoff of 1.5-fold and by statistical significance (*p* ≤ 0.01, −log_10_ *p* ≥ 2.0). Gray circles, Log_2_ FC absolute value ≤ 1.5 and −log_10_ *p* < 2.0; green circles, Log_2_ FC absolute value >1.5 and −log_10_ *p* ≥ 2.0; dark blue circles, Log_2_ FC absolute value ≤ 1.5 and −log_10_ *p* < 2.0; red circles, Log_2_ FC absolute value >1.5 and −log_10_ *p* ≥ 2.0. In total, 22 metabolites met the threshold for significance Log_2_ FC values (absolute value ≥ 1.5, *p* ≤ 0.01). Text colors of metabolite names indicate overlap among treatments. Magenta, metabolites with significant Log_2_ FC in all three stress treatments; sky blue, significant Log_2_ FC in at least two treatments; black, significant Log_2_ FC in one treatment.

The volcano plots shown in Figure 1a–c show which leaf metabolites were affected most strongly by long-duration salt, heat, or drought stress treatments, respectively. Salt stress induced accumulation of multiple amino acids—including serine, threonine, tryptophan, histidine, glutamate, lysine, tyrosine, and ornithine. In addition, several secondary compounds likely derived from amino acids were elevated—including quinic acid, pipecolic acid, and two unidentified phenolic compounds. Another prominent feature of the salt stress metabolome was accumulation of raffinose and its precursor galactinol. Amino acids (tryptophan, threonine, histidine) and raffinose pathway metabolites (raffinose and galactinol) also stood out in the heat stress metabolome profile. Unique features of the heat stress metabolome included accumulation of the sugar alcohol lactitol. Notably, citrate and trans-3-caffeoyl quinic acid were depleted relative to control samples. A unique feature of the metabolome of drought stressed leaves was a marked accumulation of hexoses (fructose, glucose, and mannose). Accumulation of raffinose and at least one aromatic amino acid was observed in all three stress metabolomes. Overall, the metabolome data included 63 compounds that were quantified in all three treatments, 69 compounds common to heat and salt profiles, 67 compounds that overlapped in drought and salt experiments, and 77 metabolites shared by heat and drought stress profiles (Supplementary Figure S2A). Hierarchical cluster analysis of metabolite data showed that control means grouped consistently with their respective treatments indicating differences among experiments (Supplementary Figure S2B). This separation of experiments likely reflected differences in growth conditions necessitated by the three stress protocols. Notably, the salt protocol included replacement of nutrients for both control and salt treated plants to avoid confounding effects of leaching. For that reason, we focused on comparisons of fold-changes relative to respective controls. Pairwise comparisons of changes in metabolites that were detected in two or more treatment profiles revealed significant correlations between stress treatments (Supplementary Figure S2C).

**Figure 2:**
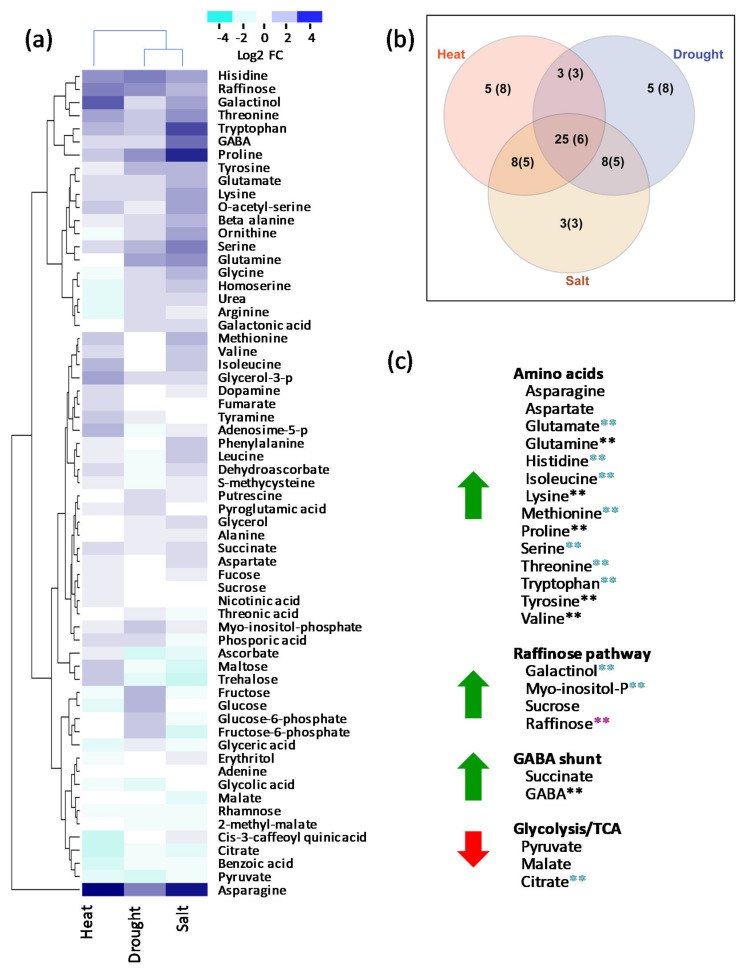
Cluster analysis of fold-changes in 63 metabolites identified in all three stress conditions. (**a**) Heat map shows log_2_ FC of 63 metabolites in the three stress treatments. (**b**) Venn diagram showing overlap of metabolites that increased or decreased in the three stress treatments. Number of metabolites that decrease is in parentheses. (**c**) Lists of selected metabolites that consistently accumulated (green arrows) or decreased (red arrows). Metabolites with significant FC changes (*p* value ≤ 0.01) are identified with asterisks. **, significant in all three treatments; **, significant in at least two stress treatments; **, significant in one treatment.

To facilitate comparisons of the metabolic impacts of long-term stress environments, we performed a cluster analysis of fold-changes in the 63 metabolites that were detected and quantified in all three stress experiments (Figure 2a). As shown in Figure 2b, the cluster analysis revealed a set of 31 metabolites that showed consistent trends across the three stress treatments (25 elevated, 6 depleted relative to controls). Notably, features of this shared set summarized in Figure 2c included 14 of the 20 protein amino acids, four compounds implicated in raffinose biosynthesis, and intermediates of the GABA shunt. By contrast, three organic acids in glycolysis and TCA pathways (pyruvate, citrate, and malate) consistently showed depletion.

### 2.3. Transcriptome Changes Underlying of Metabolic Reprogramming of Stressed Leaves

**Figure 3:**
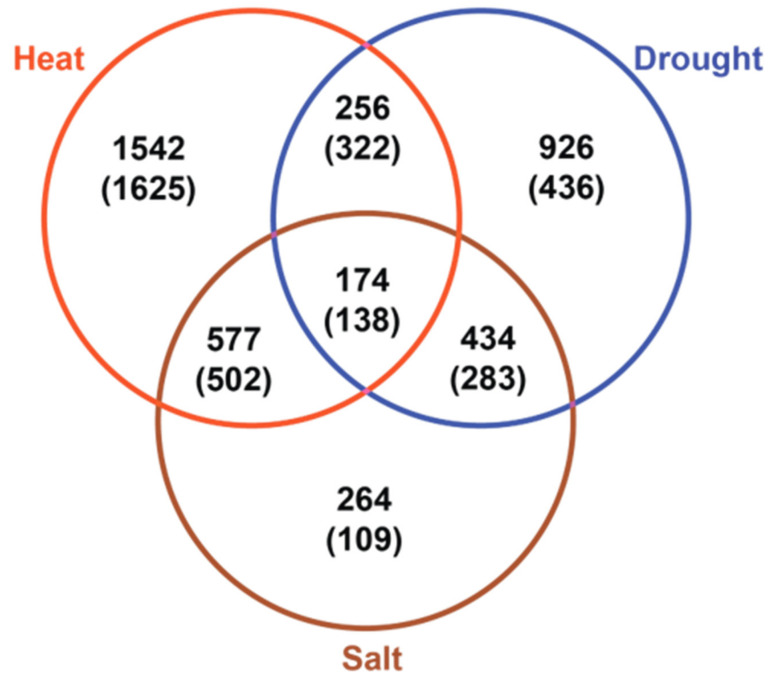
Overlap of genes differentially regulated in leaves of plants subjected to long-duration heat, drought, and salt stress. The Venn diagram shows the overlap of genes upregulated and down regulated (in parentheses) under long-term heat (red), drought (blue), and salt (brown) stress treatments.

To quantify impacts of long-term heat, salt, or drought stress on the leaf transcriptome, RNA-seq analysis was applied to leaf samples parallel to those collected for metabolite profiling. As summarized in the Venn diagram in Figure 3, the three stress treatments caused massive changes in gene expression, revealing substantial overlap between stress treatments (Supplementary Table S2–Table S10). Heat stress affected the greatest number of genes causing upregulation of 2549 genes and downregulation of 2587 genes. By comparison, salt stress affected about half as many genes including 1449 upregulated genes and 1032 downregulated genes, while the impact of drought stress was intermediate, conditioning upregulation of 1790 genes and downregulation of 1179 genes.

**Figure 4:**
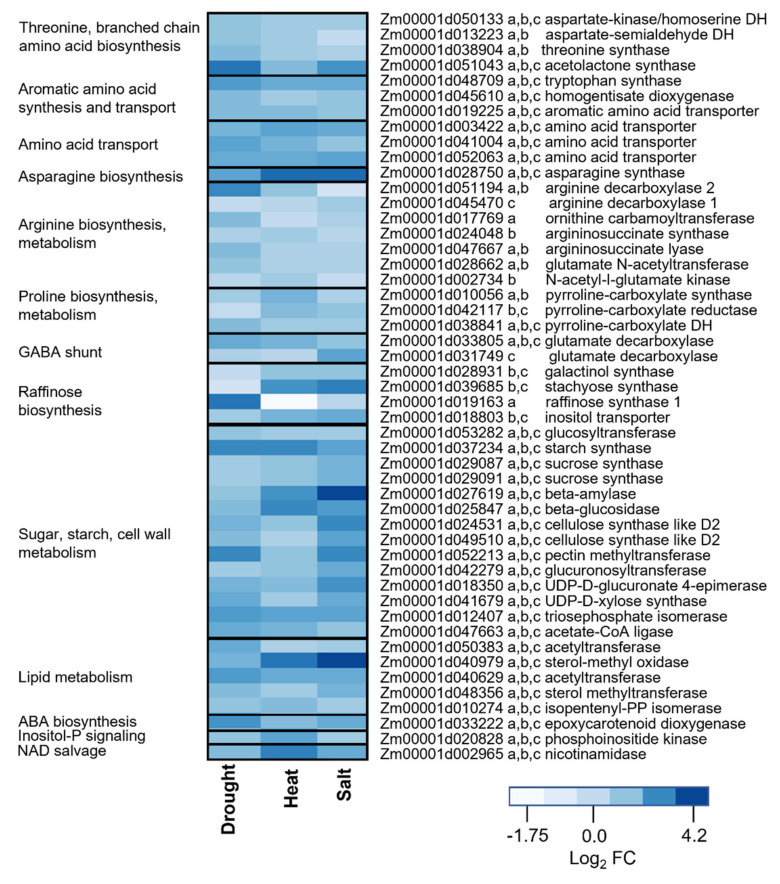
Gene expression changes in metabolic pathways upregulated by long-duration abiotic stresses. The heatmap of log_2_ FC values includes 33 genes upregulated in all three stress treatments, plus functionally related amino acid and raffinose pathway genes upregulated in at least one stress treatment. The heatmap was created using the heatmap function in R. Lowercase letters following the gene identifier indicate statistical significance (*p* ≤ 0.05) in a, drought; b, heat; and c, salt experiments. DH, dehydrogenase.

We focused on the set of 174 genes that were upregulated in all three stress treatments. GO term enrichment analysis of this group confirmed enrichment for known stress response categories including protein refolding and temperature stress functions (Supplementary Figure S3). Of particular relevance to this study, this shared set of differentially expressed genes also included 33 genes that were annotated as encoding enzymes and transporters with well-defined metabolic roles. To place the functional roles of these genes in a broader metabolic context, we augmented the shared set of 33 metabolic genes to include genes with related functional roles that were also upregulated significantly in at least one of the stress treatments. A heatmap of log_2_ (FC) of this set of genes shown in Figure 4 revealed coordinate changes in expression of genes in pathways for amino acid biosynthesis, transport, and metabolism, the GABA shunt and raffinose biosynthetic pathways. Hence, there was overall a substantial correspondence between pathways impacted by stress induced changes in the leaf transcriptome and observed shifts in metabolism. Other major pathway associations included differentially expressed genes implicated in starch and cell wall metabolism. Because complex polysaccharides were not included in our metabolite analysis, the metabolic impact of those changes was not assessed.

## 3. Discussion

Our results document metabolome and transcriptome changes that occur in leaves of maize plants grown under long-term stress environments. While stress responses of maize and other species have been studied extensively, most prior studies have focused on short-term responses typically measured within 24 h of the initial treatment [21,25]. A key goal of this study was to document metabolic and transcriptome responses to comparable, prolonged stress treatments that reflect realistic environmental conditions encountered in the field. Guo et al. [7] recently took a similar approach to analysis of interactions of drought and cold stresses.

**Figure 5:**
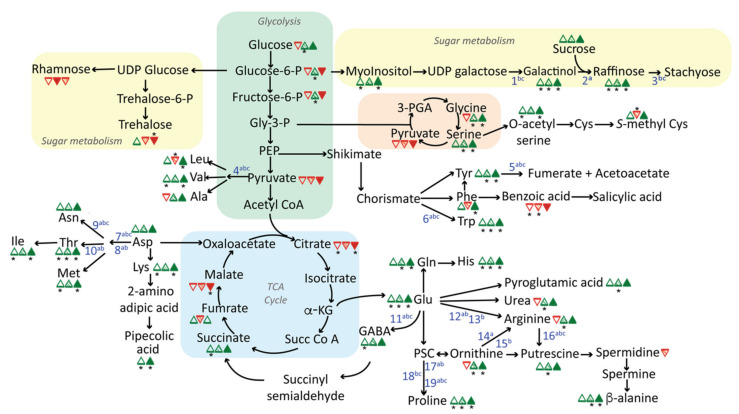
Integration of metabolite and transcriptome changes caused by drought, heat, and salt stress treatments. Open, pattern filled, and solid triangles represent metabolites changes caused by heat, drought, and salt stress, respectively. Up-modulations are in green, whereas down-modulations are in red. Asterisks indicate statistically significant changes (*p* ≤ 0.05). Raw quantification data of shown metabolites are available as Supplementary Table S1. Differentially expressed genes encoding enzymes in the relevant pathways are represented with blue numbers with superscript letters indicating statistical significance (*p* ≤ 0.05) in a, drought; b, heat; and c, salt experiments. Gene identities are 1, galactinol synthase; 2, raffinose synthase; 3, stachyose synthase; 4, acetolactone synthase; 5, homogentisate dioxygenase; 6, tryptophan synthase; 7, aspartate-kinase/homoserine dehydrogenase; 8, aspartate-semialdehyde dehydrogenase; 9, asparagine synthase; 10, threonine synthase; 11, glutamate decarboxylase; 12, glutamate N-acetyltransferase; 13, N-acetyl-l-glutamate kinase; 14, ornithine carbamoyltransferase; 15, argininosuccinate synthase; 16, arginine decarboxylase-1 and 2; 17, pyrroline-carboxylate synthase; 18, pyrroline-carboxylate reductase; 19, pyrroline-carboxylate dehydrogenase.

The metabolic context of stress-induced changes in leaf metabolites is depicted in Figure 5. At least four common themes were evident in stress metabolomes. Key features include accumulations of (1) diverse α-amino acids; (2) GABA; (3) raffinose and related carbohydrates; and (4) relative depletion of key organic acid intermediates of central metabolism (notably pyruvate, citrate, and malate). These metabolic shifts are associated with upregulation of corresponding genes in amino acid, GABA, and raffinose biosynthesis pathways indicating that the reprogramming of the metabolome under abiotic stress was to a large extent controlled at the transcriptional level. This contrasts with the pattern observed in yeast where metabolic adjustments to diverse environments occur mainly at the level of metabolic regulation rather than gene regulation [26]. In higher plants, regulation of metabolism at the transcriptome level is likely necessitated by the requirements for coordination among organs and tissues at an organismal level.

Our current results documenting the metabolome at a single leaf position of the plant (fifth leaf from the base) during the vegetative phase of development at mid-day illuminate only a small slice of the whole organism response to long-term stress environments. Potential interactions with leaf age, time-of-day, time of flowering, vegetative and reproductive phases, and other aspects of plant development are not accessed by these data. Nevertheless, common themes in the metabolic responses to long-term stress treatments clearly emerge.

### 3.1. Prominent Roles of Amino Acids in Stress Metabolomes

Among the stress-modulated amino acids, proline stands out as a well-known stress metabolite that functions as a protective osmolyte and antioxidant [5]. In this respect, proline accumulation serves as a marker confirming that stress treatments were effective. The 1-pyrrolidone-5-carboxylate precursor of proline is formed from glutamate via 1-pyrrolidone-5-carboxylate synthase [8]. Proline is also potentially derived from metabolism of arginine and ornithine [10]. The stress transcriptomes indicate upregulation of genes in the pathway from glutamate as well as pathways of arginine and ornithine metabolism. The latter are likely involved in synthesis of polyamines, which were elevated to varying degrees in all three treatments (Figure 5).

Accumulation of asparagine was associated with a striking upregulation of asparagine synthase under all three stress regimes. Indeed, this was the single most marked change observed at the transcriptome level across the three stress regimes (Figure 4). While asparagine synthesis has been implicated by several studies in stress tolerance of maize and other grasses [27,28], the precise metabolic role of asparagine metabolism in this context is much less clear than is the case for proline and arginine. One possibility is that increased asparagine synthesis in stressed source leaves is associated with an enhanced rate of nitrogen export via the phloem. Li et al. [28] linked asparagine synthase expression to increased remobilization of leaf nitrogen in reproductive stage maize plants under drought stress. While the increase in amino acid levels we observe overall is consistent with the possibility of increased protein degradation in stressed leaves of vegetative stage plants, proteins with proteolysis-related functions were not enriched among stress induced genes (Supplementary Figure S3). A more complete understanding of the roles of asparagine and other amino acids may require analysis of multiple leaf positions on the plant as well as sink organs to place changes in leaf amino acid metabolism and transport in a whole organism context.

### 3.2. GABA Shunt Activation as a Thiamin-Deficiency Workaround?

Our metabolome and transcriptome data revealed that increased capacity for GABA production was a response common to all three stresses. In contrast to other key organic acids (pyruvate, citrate, and malate), which showed a consistent pattern of depletion in stressed leaves, the GABA shunt pathway product succinate was observed to accumulate (Figure 5). Although GABA accumulation is a very common response to stress in plants, its precise roles in plant metabolism, and more so, signaling are still poorly understood [12]. We have previously proposed that adverse metabolic impacts of stress may be partly attributed to functional deficiencies in thiamin and other vitamins [14]. In this context, the GABA shunt pathway can play a critical role in ameliorating a thiamin constraint on metabolism by providing a bypass of the thiamin-dependent 2-oxoglutarate dehydrogenase enzyme of the TCA cycle [15]. This is achieved by shifting the decarboxylation reaction to the non-thiamin requiring glutamate decarboxylase [12]. Consistent with activation of the GABA shunt, one or more glutamate decarboxylase genes are upregulated in each of the three stresses (Figure 4). Interestingly, no other consistent changes in genes of thiamin biosynthesis or thiamin-dependent metabolism were seen at the transcriptome level. This does not exclude the possibility that metabolic adjustments to functional cofactor deficiencies in central metabolism occur at the level of metabolic regulation. In addition to its established role as a bypass of key steps in the canonical TCA cycle, GABA metabolism is thought to provide an important interface between C and N metabolism especially under stress conditions [12] as well as being a signaling molecule implicated in regulation of growth [29]. In addition to activity of the GABA shunt, relative depletion of pyruvate and key TCA intermediates—malate and citrate—may reflect a broad shift of carbon toward amino acid metabolism noted above. The implications of this shift for energy metabolism are not yet determined.

### 3.3. Upregulation of the Raffinose Biosynthetic Pathway

Our results indicate that a key shared metabolic response—accumulation of raffinose and raffinose precursors (Figure 2c) in stressed leaves—is associated with upregulation of multiple raffinose biosynthesis genes (Figure 4). Raffinose series sugars have well known roles as compatible solutes that confer dehydration tolerance in seeds and other plant organs [16,18,30]. In addition, galactinol and raffinose have been implicated in protection against oxidation, further extending their range of activity [17]. A recent study of the interaction of long-duration drought and cold stresses on maize showed a similar induction of the raffinose pathway by drought [7]. Although heat, salt, and drought environments are distinct, they each include—to varying degrees—a potential to impose water stress on leaf cells. Hence, biosynthesis of raffinose series sugars is likely a protective mechanism that confers tolerance to this shared stress component.

### 3.4. Multiple Signaling Mechanisms Contribute to Shared Metabolic Responses to Stress

While raffinose accumulation is a common feature of all three stress metabolomes, the stress treatments differ in their effects on expression of individual genes in the pathway. In this respect, the impact of drought stress is distinct from those of heat and salt stress. Heat and salt stresses cause coordinate upregulation of early steps in the pathway including galactinol synthase (Zm00001d028931), stachyose synthase (Zm00001d039685), and a putative inositol transporter (Zm00001d018803), whereas drought stress primarily causes upregulation of raffinose synthase (Zm00001d019163). The distinct signaling mechanisms may be rationalized because, of the three conditions, drought has the most direct impact on water status of leaf cells. Interestingly, glucose and hexose phosphates that are precursors of early raffinose pathway intermediates showed marked accumulation only under drought stress (Figure 5). One possibility is that preferential upregulation of early steps in the raffinose pathway in salt and heat stressed leaves maintains relative depletion of the hexose pool, whereas flux to raffinose precursors in drought stressed leaves is substrate driven.

Distinct signaling mechanisms are also apparent in regulation of the GABA shunt and arginine metabolism pathways. GABA accumulation is associated with differential upregulation of two paralogous genes encoding glutamate decarboxylase (Figure 4). Zm00001d033805 was upregulated most strongly by drought and heat stress, whereas salt stress acted preferentially on Zm00001d031749. Two stress-induced arginine decarboxylase paralogs exhibited a similar dichotomy with drought and heat causing induction of Zm00001d051194 and salt stress acting on Zm00001d045470. However, this pairing of drought and heat responses contrasts with the pattern exhibited by raffinose pathway genes described above where heat and salt effects were correlated. Hence, there are evidently multiple ways to achieve equivalent changes in the metabolome, highlighting the plasticity of plant metabolism.

Our results provide a basis for interpreting interactions in plants subjected to multiple stresses. In situations where the gene expression changes underlying a shared metabolic response are common to both stresses, an additive or less-than-additive interaction might be expected. By contrast, synergistic interactions are plausible where distinct signaling pathways impact the same pathway. An interesting case in that respect is raffinose biosynthesis in plants subjected to heat and drought–two stresses that frequently occur together in field environments.

Overall, our results uniquely document changes in leaf metabolism induced by long-term stress treatments that were carefully designed to reproduce realistic field environments. The shared suite of metabolic changes observed under prolonged stress includes several classes of metabolites previously identified in studies employing short duration stress treatments. This correspondence indicates that prompt metabolic and transcriptome responses to stress are largely maintained under prolonged stress. While key shared features of stress metabolomes are evidently controlled at the level of gene expression, the underlying signaling mechanisms and target genes are distinct for each of the three stresses.

## 4. Materials and Methods

### 4.1. Plant Growth Conditions

All experiments were performed with maize plants (Zea mays, inbred B73) at 24 to 35 days after planting. Plants were grown in the USDA-ARS controlled-environment greenhouse facility (Gainesville, FL, USA) between April and December, with a temperature regime of 28/23 ± 2 °C, day/night and an approximately 16 h:8 h light:dark cycle. Seeds were sown in PVC tubes, 8 cm diameter and 60 cm long, filled with Metro-Mix^®^300 (Sun Gro Horti-culture) soil containing Osmocote^®^ General Purpose Fertilizer (ICL Specialty Fertilizers), 5 g per tube. A single plant was grown in each tube, and the tubes were placed in the chambers using a complete randomized block design. For parallel metabolite and transcriptome analyses, the fifth fully expanded leaf was sampled within one hour of midday from replicate plants, growing in vegetative phase, that had been subjected to carefully controlled stress treatment regime for a period of two weeks.

### 4.2. Drought Stress

For drought stress, the water-holding capacity of each tube was determined. Drought stress was imposed beginning the fifth day after germination by ceasing watering of six replicate plants while six control replicates were watered to soil field capacity. After two weeks of treatment, leaf samples were collected for metabolomic and RNA-seq analysis from stressed and control plants. Samples were taken at 24 days after planting from the mid-blade position of the fully expanded fifth leaf counted from the base of the plant, taking one side of the midrib for transcriptomic analysis and the other side for metabolomic analysis. Drought stress was monitored by measuring relative water content. Relative water content for the sampled leaves was determined by cutting 1-cm diameter discs from the mid-position of the leaf blade, avoiding the midrib. Groups of 10 to 15 discs from each leaf sample were weighed together to determine fresh weight (FW). Discs were then floated on water in a Petri dish in light overnight, blotted dry, and reweighed to determine turgid weight (TW). Finally, discs were frozen in liquid nitrogen, freeze-dried, and weighed to determine dry weight (DW). The relative water content (RWC) was calculated using RWC% = (FW−DW) × 100/(TW−DW). Efficacy of the drought treatment was confirmed by a marked reduction in relative water content of drought treated plants (Supplementary Figure S1).

### 4.3. Heat Stress

For high-temperature stress, six replicate plants were transferred to a separate growth chamber at two weeks after germination. In the treatment chamber, temperature was raised gradually while maintaining a 16 h:8 h photoperiod as described above. Heat stress started at 30/24 °C, day/night, and increased 2 °C per day for five days, then a 37/32 °C day/night regime was maintained for 12 days. Plants were watered every three days. Control plants (six replicates) were grown as described above. Leaf samples were collected for metabolomics and RNA-seq analysis at 35 days after planting as described above.

### 4.4. Salt Stress

Proper design of salinization experiments must strive to: (1) avoid over-salinizing the soil as evapotranspiration passively raises soil salt concentration, which means irrigating liberally to achieve a high ‘leaching fraction’; (2) at the same time, avoid leaching out of nitrogen, phosphate, and micronutrients, which means adding them to the irrigation medium; (3) avoid permeabilizing root cells by an excessive monovalent/divalent cation ratio in soil water, and avoid Na^+^-induced Ca^2+^ deficiency, which both mean salinizing with a NaCl/CaCl_2_ mix.

This is what our procedure was designed to do, based on classical principles. Running a salinization experiment in a seemingly simpler way can—and often does—result in more a severe and less reproducible level of salt stress than intended, and even worse salt stress complicated by nutrient deficiencies and ion imbalances [31,32].

Fourteen plants were grown under the conditions described above. Salt stress was started at two weeks of germination. The growth tubes were drenched with 500 mL of nutrient media. The composition of the nutrient solution (mM) was: 2.5 Ca(NO_3_)_2_, 3 KNO_3_ 1.5 MgSO_4_, 0.17 KH_2_PO_4_, 0.05 Fe (C_14_H_22_FeN_3_NaO_10_), 0.023 H_3_BO_3_, 0.005 MnSO_4_, 0.0004 ZnSO_4_, 0.0002 CuSO_4_, 0.0001 H_2_MoO_4_. The pH of the solution was kept between 5.2 and 6.2, adjusted with KOH/H_2_SO_4_. For eight stressed plants, the nutrient solution was salinized in steps calculated to reduce the osmotic potential of the solution by 0.1 MPa every 2 days with NaCl/CaCl_2_ [31]. The steps were: 18 mM NaCl/3.125 mM CaCl_2_; 36 mM NaCl/6.25 mM CaCl_2_; 54 mM NaCl/9.75 mM CaCl_2_; and 72 mM NaCl/12.5 mM CaCl_2_. Plants were maintained at −0.4 MPa osmotic potential for 8 days. Non-salinized control plants were drenched with the same volume of nutrient solution without NaCl/CaCl_2_. Leaf samples were collected at 24 days after planting for metabolomics and RNA-seq analysis as above.

### 4.5. Metabolite Analysis

Metabolite profiling was performed by GC-MS as described by Suzuki et al. [33]. Briefly, ground frozen leaf tissue was homogenized at 70 °C for 15 min in 300 μL of methanol followed by addition of 200 μL of chloroform and 300 μL of water. The polar phase was collected and dried under vacuum. The residue was derivatized at 37 °C in 50 µL of 20 mg mL^−1^ methoxyamine hydrochloride in pyridine incubating for 120 min followed by a 30 min treatment with 50 µL of MSTFA. Analyses were performed using a Leco Pegasus HT TOF-MS gas chromatograph (Leco Inc, St. Joseph, MI, USA) with coupled time-of-flight mass spectrometer. Samples were injected using a Gerstel Multi-Purpose autosampler (Gerstel GMbH, Mülheim an der Ruhr, Germany). A 30 m DB-35 gas chromatography column was used with helium carrier gas flow rate of 2 mL s^−1^. A 230 °C injection temperature was used with transfer line and ion source set to 250 °C. The oven temperature (initially 85 °C) was increased at a rate of 15 °C min^−1^ to a final temperature of 360 °C. Following a delay of 180 s for solvent mass spectra were recorded with 20 scans s^−1^ over a 70–600 *m/z* range. Chroma TOF 4.5 (Leco Inc., St. Joseph, MI, USA) and TagFinder 4.2 software [34,35] were used to evaluate chromatograms and mass spectra.

### 4.6. RNAseq

RNA-seq analysis was performed as described by Mimura et al. [36] and Suzuki et al. [33]. Briefly, leaf samples were ground in liquid nitrogen and stored at −80 °C. Total RNA was extracted from a spatula scoop of frozen ground leaf sample using the Plant RNeasy kit (Qiagen). RNA-seq libraries were constructed using TruSeq^®^ Stranded Total RNA LT-with RiboZero™ Plant (Illumina). Illumina sequencing was conducted with a HiSeq instrument at UF ICBR service core facility. Unidirectional reads were mapped to the maize B73 v2 assembly and analyzed using TopHat, Cufflinks, and Cuffdiff [37] hosted on the Galaxy platform at UF HPC.

### 4.7. Statistical Analysis

Principal component analysis (prcomp) and hierarchical clustering (hclust) of metabolite and transcriptome fold-change data were performed using standard methods in R (r-project.org). Heat maps were created using the heatmap function in R.

## Data Availability

The RNA-seq data presented in this study are openly available in the National Center for Biotechnology Information SRA database under bio-project accession PRJNA368967. Metabolite data presented in this study are available in Supplementary Table S1.

## Supplementary Materials

The following are available online at https://www.mdpi.com/article/10.3390/metabo11110797/s1, Supplementary Figure S1: Relative water content of drought stressed leaves, Supplementary Figure S2: Common metabolites measured in three different stress conditions, Supplementary Figure S3: GO category enrichment of genes upregulated in heat, drought and salt stressed leaves, Supplementary Table S1: Metabolite data, Supplementary Table S2–S9: RNA-seq FPKM and fold-change data for heat, salt and drought experiments, and Supplementary Table S10: GO term enrichment analysis.
