# Supplementary material for: A Core Metabolome Response of Maize Leaves Subjected to Long-Duration Abiotic Stresses
Source: Metabolites. 2021 Nov 22;11(11):797. doi: 10.3390/metabo11110797 (PMC8625080; doi:10.3390/metabo11110797)
Supplement: Supplementary file 1 [file metabolites-11-00797-s001.zip › metabolites-1410411-supplementary.pdf]

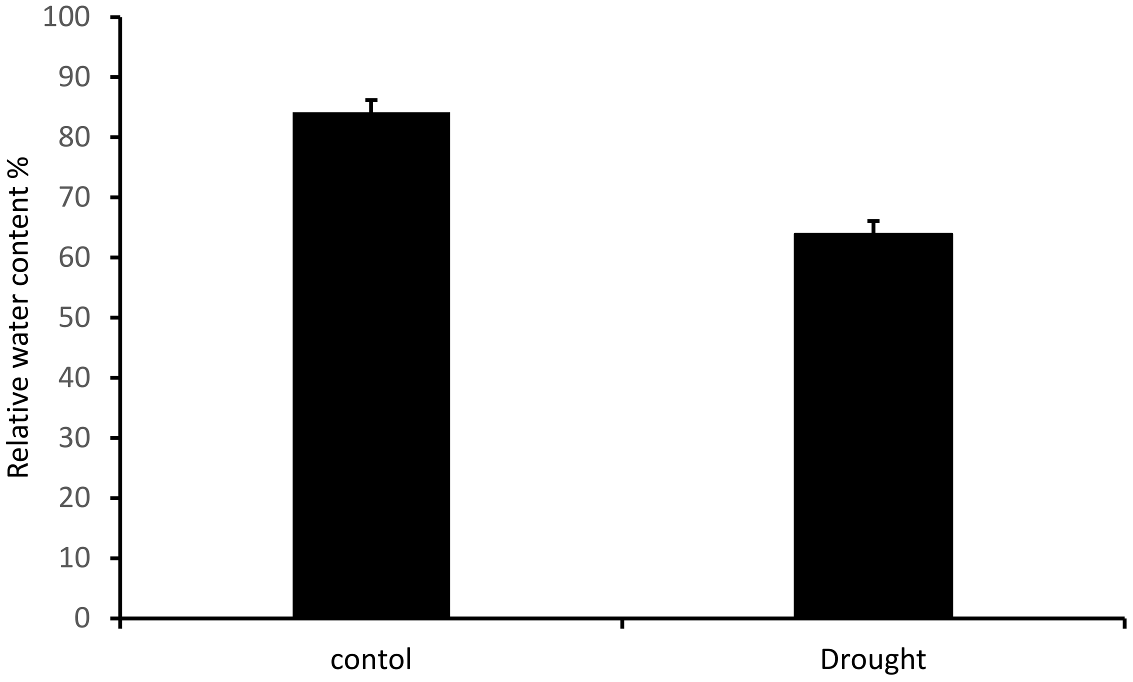

Supplementary Figure S1. Mean relative water content (RWC%) of drought stressed and control leaves. Data is showing average of six replicates of control and drought stressed plants.

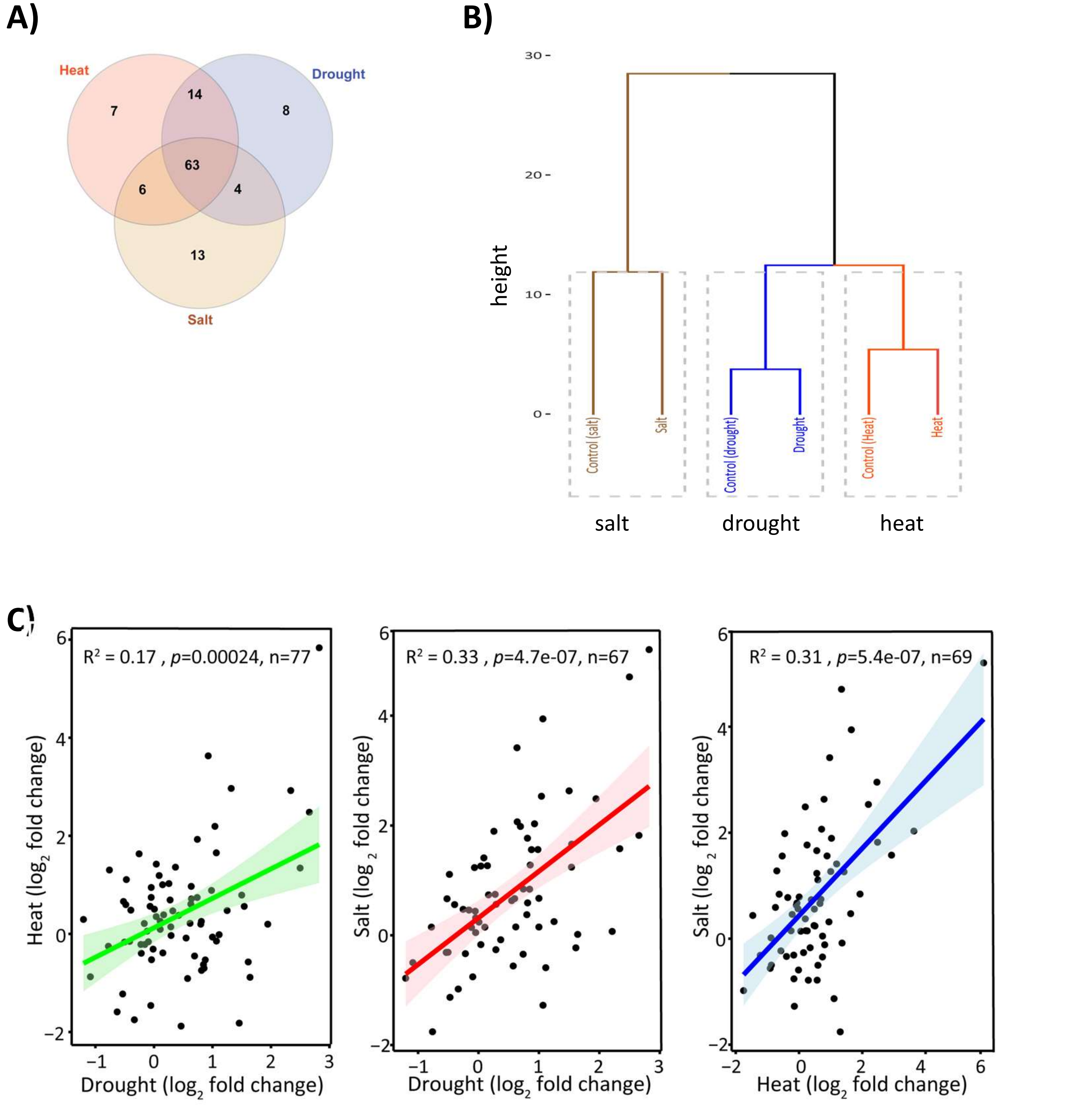

Supplementary Figure S2: Common metabolites and there pattern in three different stress conditions **A)** Venn diagram representing number of shared and unique metabolites in three stress conditions B)Co-relation between various stresses. **B)** Hierarchical cluster analysis of control and treatment values for 63 metabolites detected in salt, drought and heat experiments. **C)** Pierson correlation graph represent co-relation between log_2 fold change value of common metabolite in heat and drought stress; salt and drought stress and salt and heat stress..

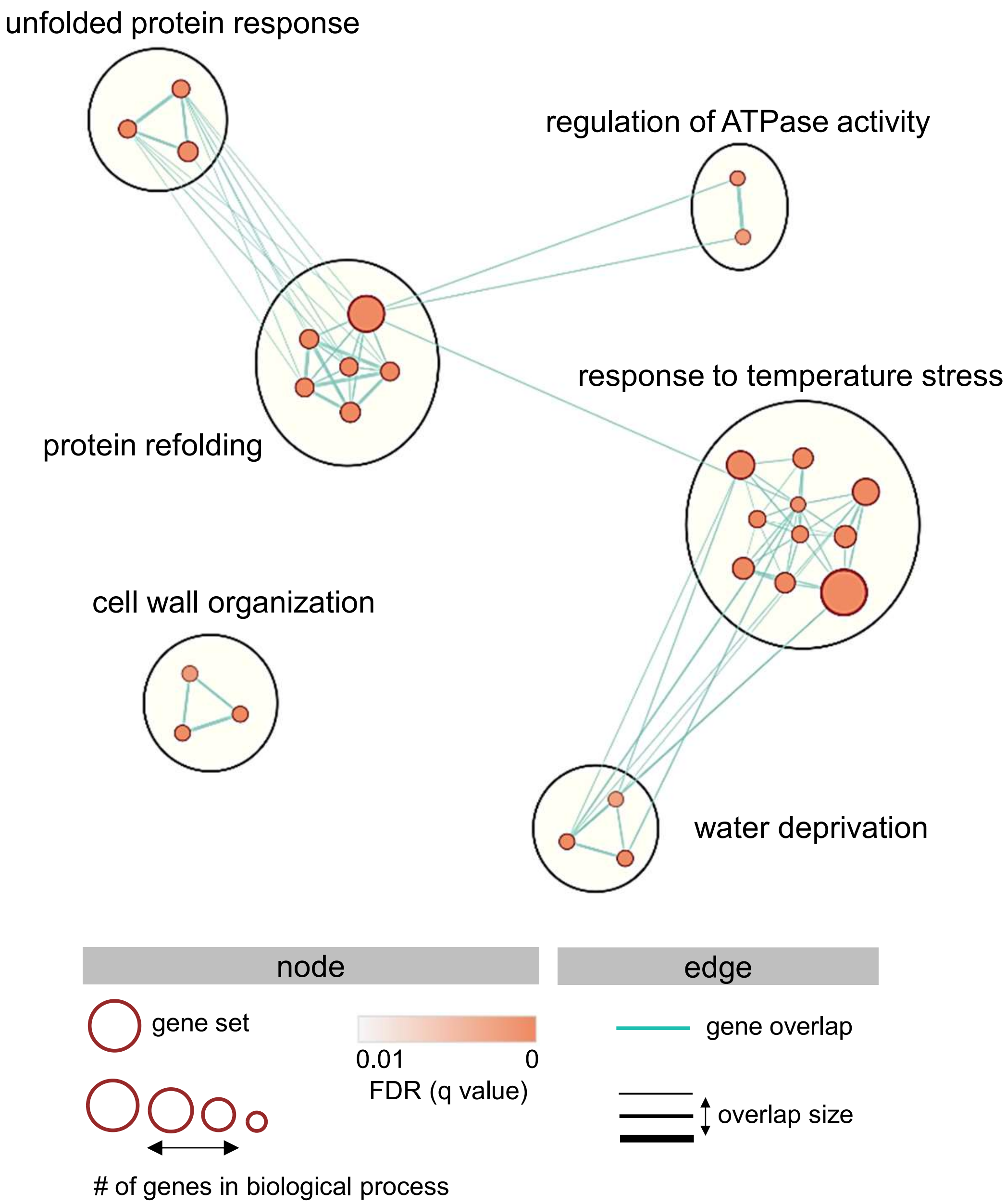

Supplementary Figure S3 GO category enrichment of genes upregulated in heat, drought and salt stressed leaves. GO term enrichments were determined for 174 genes upregulated in all three stress treatments using the Pathway Enrichment tool (Reimand et al., 2019). No enriched categories were detected for genes down regulated in all three treatments.

Reference:
Reimand, J., Isser, R., Voisin, V., Kucera, M., Tannus-Lopes, C., Rostamianfar, A., Wadi, L., Meyer, M., Wong, J., Xu, C., Merico, D., and Bader, G. D. Pathway enrichment analysis and visualization of omics data using g:Profiler, GSEA, Cytoscape and EnrichmentMap. Nature Protocol 14 (2): 482-517.
